# Parasitic infection may be associated with discordant responses to QuantiFERON and tuberculin skin test in apparently healthy children and adolescents in a tuberculosis endemic setting, Ethiopia

**DOI:** 10.1186/1471-2334-13-265

**Published:** 2013-06-05

**Authors:** Liya Wassie, Abraham Aseffa, Markos Abebe, Michael Z Gebeyehu, Martha Zewdie, Adane Mihret, Girum Erenso, Menberwork Chanyalew, Hiwot Tilahun, Lawrence K Yamuah, Peter Andersen, Mark T Doherty

**Affiliations:** 1 Armauer Hansen Research Institute, Jimma Road, P.O. Box: 1005 Addis Ababa, Ethiopia; 2 Infectious Disease Immunology, Statens Serum Institute, Artillerivej 5 2300 S, Copenhagen, Denmark; 3 Current Address: GlaxoSmithKline, 68 Nykær, Copenhagen, Denmark

**Keywords:** Latent tuberculosis, QuantiFERON, TST, Adolescents, Children, Parasites

## Abstract

**Background:**

*M. tuberculosis* remains one of the world’s deadliest pathogens in part because of its ability to establish persistent, latent infections, which can later reactivate to cause disease. In regions of the globe where disease is endemic, as much as 50% of the population is thought to be latently infected, complicating diagnosis and tuberculosis control. The tools most commonly used for diagnosis of latent *M. tuberculosis* infection are the tuberculin skin test and the newer interferon-gamma release assays, both of which rely on an antigen-specific memory response as an indicator of infection. It is clear that the two tests, do not always give concordant results, but the factors leading to this are only partially understood.

**Methods:**

In this study we examined 245 healthy school children aged from 12 to 20 years from Addis Ababa, a tuberculosis-endemic region, characterised them with regard to response in the tuberculin skin test and QuantIFERON™ test and assessed factors that might contribute to discordant responses.

**Results:**

Although concordance between the tests was generally fair (90% concordance), there was a subset of children who had a positive QuantIFERON™ result but a negative tuberculin skin test. After analysis of multiple parameters the data suggest that discordance was most strongly associated with the presence of parasites in the stool.

**Conclusions:**

Parasitic gut infections are frequent in most regions where *M. tuberculosis* is endemic. This study, while preliminary, suggests that the tuberculin skin test should be interpreted with caution where this may be the case.

## Background

Tuberculosis (TB) is one of the infectious diseases that claims the most lives globally with annual estimates of approximately two million deaths and more than 9 million new cases [1]. More than 90% of new cases occur in the developing countries [2], and Africa is particularly hard-hit with an estimated incidence rate of 363 and a prevalence of 475 per 100,000 population [1]. Tuberculosis is also a national health priority in Ethiopia, since in that country, it is a leading cause of morbidity, the 3rd most frequent cause of hospital admission and 2nd most frequent cause of death after malaria [3]. Tuberculosis affects both sexes and all ages, though in developing countries the active disease is primarily diagnosed in those between 15 and 45 years of age [1,4]. After exposure to the TB-causing bacillus, *Mycobacterium tuberculosis* (*M. tuberculosis*); roughly a third of the exposed individuals will acquire the infection [5]. Of these infected individuals, only 10% will present with clinical manifestations, while the majority remain latently infected [5-7]. Although a number of studies have been conducted to understand the mechanism underlying latency [5,8,9], the age at which this infection is acquired in life (and thus how much TB is actually reactivation of latent infection versus primary infection) is still debatable and data on how apparently healthy community controls behave immunologically at the early stage of such infection is still limited.

We therefore recruited a cohort of apparently healthy individuals between the age of 12 and 20 years from schools in Addis Ababa, the capital city of Ethiopia: a region where TB is endemic and transmission presumably frequent. As there is no gold standard for diagnosis of latent TB infection (LTBI), we compared the two most commonly-used immunodiagnostic tools used to identify LTBI, the tuberculin skin test (TST) and QuantiFERON TB Gold In-Tube (QFT-GIT). We also characterised this cohort with regard to factors which are thought to be relevant for tuberculosis disease, such as socioeconomic status, body mass index (BMI) scores, etc., as well as for expression of several genes previously shown to be associated with responses to *M. tuberculosis* infection. We found a generally fair correlation between the TST and QFT-GIT test, but the number of discordant results was noticeably higher in participants from poorer, government-supported schools. Of the factors analysed, parasitic infection, as assessed by the presence of parasites in the stool, appeared to be associated with a positive QFT-GIT result combined with a negative TST result.

## Methods

### Study design and participants

This manuscript reports on a cross-sectional study, conducted between March and September 2009, involving a total of 245 apparently healthy school children and adolescents, between the age of 12 and 20 years. All students lived and attended schools in Addis Ababa, the capital city of Ethiopia, where the incidence rate of TB is high and BCG vaccination is routinely administered at birth. All participants were HIV-negative and those who had a history of chronic illness, anemia or asthma/atopy were excluded from the study during recruitment after clinical/physical examination. In Ethiopia the basic education system involves enrollment around the age of 7, although children frequently enroll below this age. For practical reasons, children younger than 12 could not be investigated due to among others the reluctance of parents and guardians to allow testing and blood draws from younger healthy children.

In this study, participants were recruited from 7 different schools, (3 private and 4 government) located in three different sub-cities (Kolfe-Keranio, Lideta and Nifas-Silk Lafto), city administrative units of Addis Ababa, constituting a population between 200,000 and 500,000 [10]. The study sites were selected based on close proximity to Armauer Hansen Research Institute (AHRI) laboratory, to avoid the risk of poor performance of laboratory procedures on the collected specimens due to handling delays, of which the different schools were selected randomly. Although basic education is similar in most schools in Ethiopia, the number of pupils attending per class and the resources vary depending on the type of school, where number of pupils per class in private schools is much smaller compared to government schools. In this study, the rationale for the selection of different school types was to include different levels of crowding and other possible risk factors of *M. tuberculosis* infection and transmission, which could serve as proxies for differences in the socio-economic situation of participants.

The socio-demographic characteristics of the study participants and their physical and clinical status were examined and recorded on questionnaires by the study physician (Table 1). This study was conducted after receiving ethical approval from the Armauer Hansen Research Institute/All Africa Tuberculosis/Leprosy Rehabilitation and Training Center (AHRI/ALERT) Ethics Review Committee and the National Research Ethics Review Committee at the Ministry of Ethiopian Science and Technology. Only participants for whom written informed consent was given by parents or guardians and who themselves had assented or who were above 18 years and could give their own consent, participated in the study.

**Table 1:** Description of socio-demographic features of the study participants (N= 245)

**Variables**	**Study participants**
**Age (yrs)**	
Mean (SD)	14.8 (1.7)
Median (Range)	15 (12–20)
**Age category**	
12-15 yrs: N (%)	168 (68.6)
16-20 yrs: N (%)	77 (31.4)
**Sex**	
Male: N (%)	112 (45.7)
Female: N (%)	133 (54.3)
**BMI (Kg/M**^**2**^**)**	
Mean (SD)	17.3 (2.7)
Median (Range)	16.9 (12–28)
BMI category*	
**Boys**	
Underweight (<20): N (%)	109 (44.5)
Normal (20–30): N (%)	3 (1.2)
Overweight (>30): N (%)	0 (0)
**Girls**	
Underweight (<18): N (%)	71 (29.0)
Normal (18–32): N (%)	62 (25.3)
Overweight (>32): N (%)	0 (0)
**School type**	
Government: N (%)	153 (62.4)
Private: N (%)	92 (37.6)
**Family size**	
Mean (SD)	5.6 (2.0)
Median (Range)	5 (1–14)
**Family size category**	
1-6: N (%)	178 (73.0)
7-14: N (%)	66 (27.1)
**Education status of participants’ families**	
**Father**	
Illiterate: N (%)	23 (11.4)
Primary education: N (%)	42 (20.9)
Secondary education: N (%)	77 (38.3)
Beyond secondary education: N (%)	59 (29.4)
**Mother**	
Illiterate: N (%)	43 (18.7)
Primary education: N (%)	82 (35.7)
Secondary education: N (%)	63 (27.4)
Beyond secondary education: N (%)	42 (18.3)

*BMI classification is based on the CDC’s categorization [16].

### Sampling

Approximately 6 ml of peripheral venous blood was drawn from all eligible participants, of which 3 ml was used for QuantiFERON assay (Cellestis, Australia) and the remaining blood aliquoted into PAXgene blood RNA tubes (PreAnalytiX, QIAGEN) for analysis of cytokine mRNA expression. After collection, all blood specimens were transported to the AHRI laboratory at ambient temperature, whereupon the QuantiFERON tubes were incubated at 37°C immediately upon arrival and the PAXgene tubes were kept at room temperature for about 2 hours prior to processing. In addition to blood, stool specimens were also collected from all participants to investigate a possible role for parasites as risk factors for LTB infection and laboratory examinations were carried out using direct smear microscopy as well as the formalin ether concentration technique as described earlier [11].

### QuantiFERON and tuberculin skin test

Approximately 1 ml of venous blood was aliquoted into each of three separate tubes of the QuantiFERON-TB Gold In Tube kit (QFT-GIT) (Cellestis, Australia): one tube containing only heparin, a negative control (Nil), another tube containing phytohaemagglutinin (PHA), a positive control (Mitogen) and the third tube containing overlapping peptides of ESAT-6, CFP-10 and TB7.7 (*M. tuberculosis* antigen). Assays were performed according to the manufacturer’s instruction, i.e., after blood drawing, tubes were shaken vigorously and incubated at 37°C (within 3 hours of blood collection) for about 20 hours at which time plasma was collected from each tube and stored at −20°C until assayed by ELISA. The level of IFN-γ was measured using the QFT-GIT ELISA In-Tube Kit (Cellestis, Australia) and test interpretation and results were analyzed using QuantiFERON-TB Gold Analysis Software (Version 2.50, Cellestis, Australia). A test for *M. tuberculosis* infection is considered positive if the amount of IFN-γ is ≥ 0.35 International Units (IU)/mL for (TB Antigen-Nil) and the test is valid if the (Mitogen-Nil) response is ≥ 0.50 IU/mL and/or (TB Antigen-Nil) is ≥ 0.35 IU/mL, according to the manufacturer’s instruction.

In addition to the QFT-GIT assay, a TST was done for all study participants, by the same study team, just after blood was drawn for the QFT-GIT assay. Briefly, 0.1 ml of 2 T.U. of Tuberculin PPD RT 23 (SSI, Denmark) was administered intradermally in the middle third of the lateral surface of the forearm and the transverse induration diameter was measured after 48–72 hours using the ball-point method to determine edges. A positive test interpretation was recorded using a 10 mm cut-off, according to the CDC TST classification for individuals living in highly TB-endemic settings [12].

### RNA extraction, cDNA synthesis and quantitative Real-time PCR (qRT-PCR)

Extraction of RNA, cDNA synthesis and subsequent real-time PCR were performed as described earlier [13,14]. Briefly, RNA was extracted from whole blood using the PAXgene Blood RNA Kit (PreAnalytiX, QIAGEN) according to the manufacturer’s instructions. The mRNA was reverse transcribed into cDNA, using the Omniscript Reverse Transcription Kit (QIAGEN, Germany) with the oligo(dT) primers (Promega, USA) according to the manufacturers’ instructions. The concentration of cDNA was further quantified using a GeneQuant spectrophotometer (Amersham Biosciences, UK), adjusted to the same level, aliquoted and stored at −20°C until assayed by quantitative real-time PCR (*qRT-PCR*).

In this study, we measured mRNA expression of three different cytokines; IFN-γ, IL-4 and its functional antagonist and splice variant, IL-4δ2, and a regulatory T-cell (T_Reg_)-associated transcription factor, fork-head box P3 (FoxP3), in whole blood using the Rotor Gene-3000 system (Corbett Research, Australia). The *qRT-PCR* was carried out in a total volume of 12.5 μl, containing 1 μg of starting cDNA template using QuantiTect Probe RT-PCR Kit (QIAGEN, Germany) as described earlier [13]. Briefly, primers (Alpha DNA, Canada) and *Taq*Man probes (Applied Biosystems, USA) were designed to span exon-intron junctions to prevent amplification of genomic DNA and to result in amplicons of fewer than 150 base pairs to enhance the efficiency of PCR amplification. Probes were labeled with the reporter dye molecule, FAM (6-carboxy-fluorescein; emission λmax = 518 ηm) and a quencher dye molecule, TAMRA (6-carboxytetramethyl-rhodamine; emission λmax = 582 nm) at the 5′ and 3′ end, respectively. The final concentration of primers and probes in each reaction was optimized between 200 nM and 500 nM. All the PCR reactions were run in duplicate and non-template controls were included in all the assays together with the samples, to test for contamination of reagents; and all the mixes were prepared using a Corbett sample preparation robot (Corbett Research, Australia).

Reaction efficiencies were derived from serial dilutions of cloned PCR product (standard copies of a specific gene) and if variation between duplicates varied by more than 10% the runs were repeated. Gene copy numbers of cytokine mRNA were quantified against a standard curve, normalized to a housekeeping gene, human acidic ribosomal protein (HuPO) that has been extensively validated earlier [13]. In cases where the standard copies were not available, data were analyzed using the 2(−delta delta C(T)) method as previously described [15].

### Data analysis

The data presented here were analyzed using STATA Data Analysis Software (Version 8.2, USA) and GraphPad Prism version 4.00 for Windows (GraphPad Software, San Diego California USA). Analysis of contingency tables and correlation between the different groups assumed different statistical approaches including logistic regression, multivariate analysis and chi-square test. Analysis of cytokine mRNA expression assumed one-way ANOVA (Kruskal-Wallis test) with Dunnett’s post test. In all instances, a P-value < 0.05 was considered statistically significant.

## Results and discussion

### Characteristics of study participants

Although schools were randomly picked, a slightly larger number of pupils were recruited from government compared to private schools (Table 1). Although all participants appeared to be apparently healthy, the majority of the participants (73%, 180/245) fall under the lower limit of BMI (Table 1), following the CDC categorization [16]. There also appeared to be a difference in the percentage of participants who were underweight by gender. However, these differences must be treated with caution, as both normal weight and sexual dimorphism can vary by ethnicity, and there are to our knowledge no standardized BMI values in use as reference for Ethiopians.

Other factors such as family size and education level of participants’ families were assessed, as possible proxy markers for crowding or socio-economic status, both identified in the past as possible risk factors for *M. tuberculosis* infection. The median family size of the participants was five, a clear majority (73%) had a family size below six (Table 1); and most of the participants’ families had reached at least the secondary level of education (9–12 grade). This relatively high level of academic achievement can probably be attributed to the fact that participants were recruited from the capital city, Addis Ababa, where the majority of the city dwellers are literate; in contrast to the 2007 estimate by the United Nations Development Program, which reported the rate of literacy in the general population of Ethiopia to be around 35.9% [17].

In addition to the demographic features, the study participants were also examined for signs of parasitic infection in their stool. In this study we defined parasitic infection as detecting one or more of the following parasites in the stool specimens: *Giardia lamblia*, *Ascaris lumbricoides*, *Hookworm spp.*, *Strongyloides stercoralis*, *Trichuris trichuria*, *Enteroboius vermicularis*, *Taenia spp.*, *Hymenolopis nana*, *Schistosoma mansoni* or trophozoite stage of *Entamoeba histolytica*. Although relatively few abdominal complaints such as nausea and cramp were reported by the participants, the overall rate of confirmed parasitic infections was about 20% (49/245) (Table 2), with the proportion of infection being slightly skewed towards females (55%). In addition, there was a strong association between school type and risk of parasite infection (P = 0.01), where the proportion of parasite infestations was significantly higher (above 75%) among participants attending government schools (Table 2) compared to private schools; possibly indicating more infections are acquired in the poorer communities. The percentage level of individual parasitic infections is detailed in Table 2, though there were also a few cases of double infections (~12%) of *Giardia lamblia* with *Hookworm spp.* or *Hymenolopis nana* or *Entamoeba histolytica* trophozoite; *Ascaris lumbricoides* with *Trichuris trichuria* or *Hymenolopis nana* and *Trichuris trichuria* with *Hymenolopis nana*.

**Table 2:** Parasite burden among study participants (N= 245)

**Parasites diagnosed**	**Study participants: N (%)**
Total proportion of participants with parasitic infestation*	49 (20)
Proportion of participants with single parasite infestation (total)	43 (87.8)
*Giardia lamblia*	18 (36.7)
*Ascaris lumbricoides*	5 (10.2)
*Hymenolepis nana*	5 (10.2)
*Trichuris trichiura*	5 (10.2)
*Taenia spp.*	4 (8.2)
*Entamoeba histolytica* trophozoite	4 (8.2)
*Enterobius vermicularis*	1 (2.0)
*Hookworm sppc*	1 (2.0)
Proportion of students with parasites/school type**	
Government	38 (77.6)
Private	11 (22.4)
Proportion of participants with parasites by gender	
In males	22 (44.9)
In females	27 (55.1)

* Includes single and double concurrent parasite infections.

** School type was used as a proxy for socio-economic class. Private schools are fee dependent, whereas government schools rely entirely on government support and are free to attend.

### Comparison of the tuberculin skin test and the QuantiFERON™ test in the study population

The rate of LTBI among study participants was estimated using the TST, a well-established tool for immunodiagnosis of *M. tuberculosis* infection and the QFT-GIT, a newer and more specific test [18,19]. The overall rate of LTBI among study participants was approximately 11% by TST and 21% by QFT-GIT. The median induration size of the TST was 0 mm, range (0–33 mm) and a substantial majority (86%) had a TST induration size of less than 5 mm, of whom 11% were identified as positive by QFT-GIT. More than 90% (24/26) of the cases who were positive by TST were also identified as positive by QFT-GIT. The level of agreement between QFT-GIT and TST was moderate (κ-value = 0.56; P < 0.0001). We also saw a significant association between TST induration size and QFT-GIT response, where a larger induration size correlated with a higher likelihood of a positive QFT-GIT response (P < 0.0001). Among our participants, eight individuals had a TST induration size of 15 mm or above and all were detected as positive by QFT-GIT.

### Correlations between socioeconomic indicators and positivity in the tuberculin skin test or QuantiFERON test

We looked at the impact of other factors such as sex, BMI, TB contact history, education level of participants’ families and family size of participants on acquisition of *M. tuberculosis* infection as assessed by TST or QFT-GIT and no apparent association was observed between these factors and a positive test outcome. In this study, we had only few individuals (19/245) who had a history of TB in their families and thus of possible contact; of whom 6 were positive by either of the tests, and of these, 4 were positive by both TST and QFT-GIT.

However, when we began to compare responses divided by the type of school from which participants were recruited, we began to see divergent results. There was a significantly larger proportion of participants recruited from government schools having a positive TST response (Table 3) and a two by two chi-square table comparison further indicated that pupils from government schools were at higher risk of becoming positive by TST compared to those recruited from private schools [OR= 2.8; 95% CI (1.01, 7.62)]. On the other hand, although there was no significant association between school type and QFT-GIT response, a slightly larger proportion of participants from government schools were also positive by QFT-GIT (p-value = 0.64) (Table 3).

**Table 3:** Proportion of QFT-GIT and TST positive responses with respect to school types

**Test result**		School type**: N (%)	
		**Private**	**Government**
**QFT**	**Negative**	69 (75.0)	118 (77.1)
	**Positive**	17 (18.5)	34 (22.2)
	**Indeterminate**	6 (6.5)	1 (0.7)
**TST**^¶^	**Negative**	87 (94.6)	132 (86.3)
	**Positive**	5 (5.4)	21 (13.7)*

^¶^TST measured at 10 mm cut-off; 85%CI: et al. 2012ST positives among Ethiopian adults recruited from medical schools.

* P-value = 0.0413; ** School type was used as a proxy for socio-economic class. Private schools are fee dependent, whereas government schools rely entirely on government support and are free to attend.

**Figure 1:**
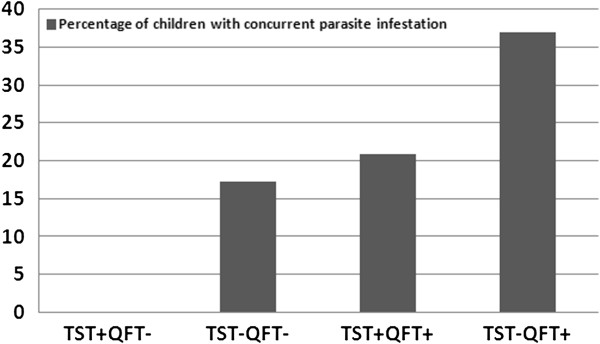
**Association between parasite infestation and TST and QFT-GIT response.** The figure shows the study cohort segregated by their response in the TST and QFT-GIT tests and then analyzed for the percentage in each subgroup with a concurrent parasite infestation as identified by stool sampling. The X-axis indicates category of immunodiagnostic test results as (‘-’ = negative and ‘+’ = positive) and the Y-axis indicates the percentage of children with at least one confirmed parasite infection.

It can be seen that government schools, which have large class sizes and generally cater to a population with lower socioeconomic status, have a higher rate of students with concurrent parasitic infestations (Table 2), which may impact the test outcomes. This hypothesis was supported by an analysis of parasite infection and test outcomes, which indicated a significant association between parasite infestation and a positive QFT-GIT response (P-value = 0.02), but not with TST [OR = 1.3; 95% CI (0.5, 3.1)]. From a chi-square analysis, individuals infected with parasites were at a higher risk of having a positive QFT-GIT response compared to non-parasite infected individuals [OR = 2.2; 95% CI (1.2, 4.3)]. In addition, the proportion of parasite infestation among participants with discordant test results also indicated an association between a positive QFT-GIT response and a parasite infection, as it was 37% in TST- QFT-GIT+ individuals compared to nil among those whose results were TST+ QFT-GIT- (Figure 1).

### Analysis of cytokine mRNA expression among TST and QuantiFERON positive and negative groups

**Figure 2:**
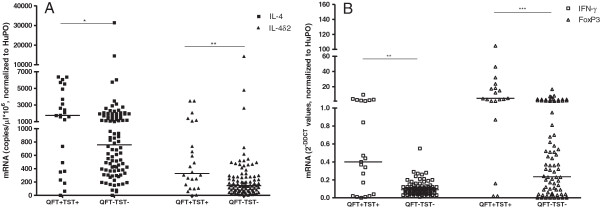
**Pattern of cytokine mRNA expression among participants segregated according to QFT-GIT and TST results.** Data are from whole blood, showing mRNA levels, assessed by qRT-PCR, normalized and expressed relative to the housekeeping gene, human acidic ribosomal protein (HuPo) as fold values. Each point represents the mean of duplicate experiments from a single individual. In both figures, horizontal lines indicate medians and *shows a p-value = 0.0237, ** shows p-values = 0.0035 and 0.0027 for Figure **A** and **B**, respectively and *** shows a p-value < 0.0001.

Earlier studies in Ethiopia in adults have suggested that cytokine mRNA expression for IL-4, IL-4δ2 and IFN-γ differs between apparently healthy individuals with and without LTBI [14,20] while other studies have implicated the regulatory cell marker FoxP3 as a potential marker of immune status in *M. tuberculosis* infection [21]. When we compared expression of these genes between individuals who were either QFT-GIT and TST positive to those negative for both tests, we found clear and significant differences (Figure 2). Those individuals positive for both tests had significantly higher levels of expression of mRNA for IL-4, a Th2 cytokine, but this was balanced by higher levels of mRNA for the IL-4 antagonist, IL-4δ2 and the Th1 cytokine IFN-γ. This is the same cytokine mRNA profile previously seen in healthy Ethiopian adults with presumed latent infection [20]. Analysis of those positive for either the QFT-GIT or the TST, without regard to the other test, showed the same effect: a positive result in either test was associated with higher expression of all 4 genes (data not shown). This is perhaps not surprising, given the degree of concordance between the two tests.

**Figure 3:**
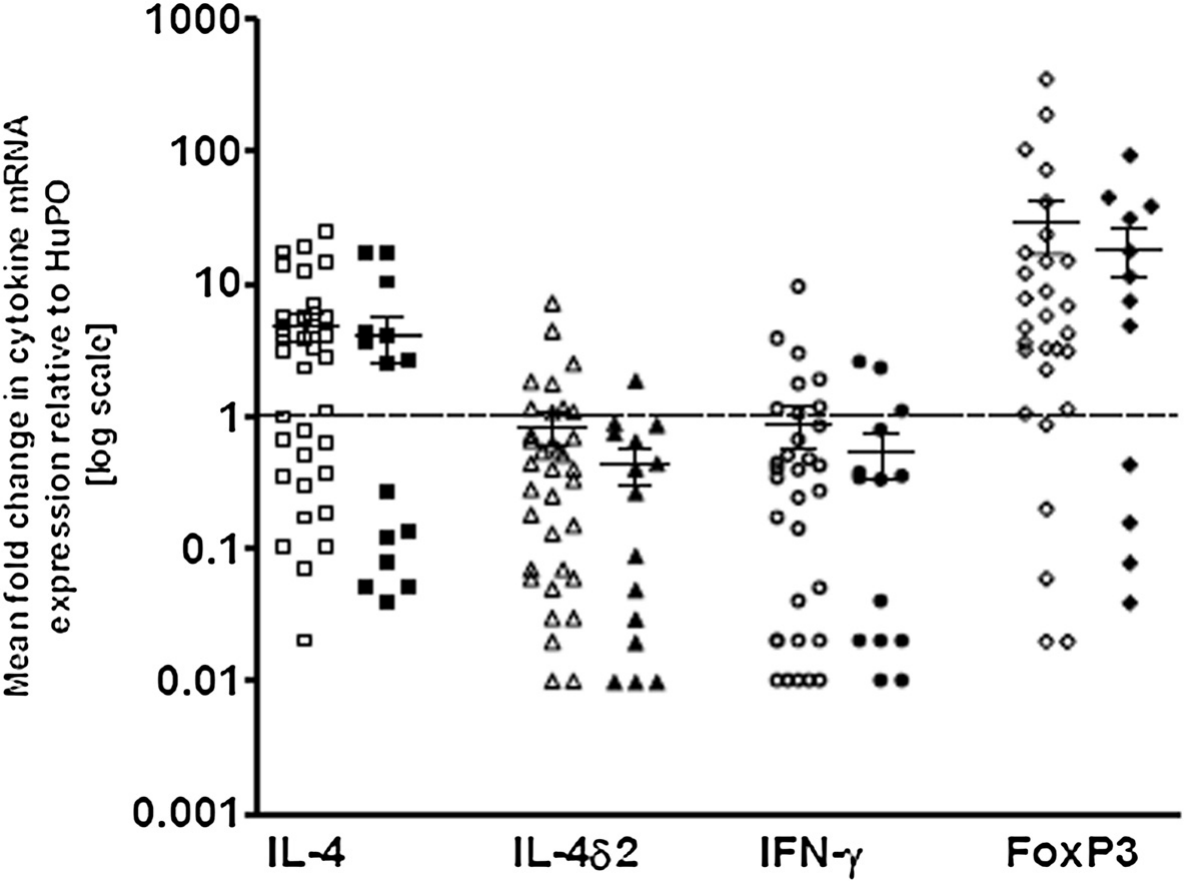
**Cytokine mRNA expression pattern among QFT-GIT-positive individuals, without (n = 36) and with concurrent parasitic infestation (n = 15).** Data are from whole blood, showing mRNA levels, assessed by qRT-PCR, normalized and expressed relative to the housekeeping gene, human acidic ribosomal protein (HuPo). Empty dots indicate non-parasite infected and filled dots indicate parasite infected individuals. Each point represents the mean of duplicate experiments from a single individual and the median and interquartile values are shown by horizontal lines across the data points for each group.

To determine if parasite infection affected expression of these cytokines, in participants with presumed LTBI, we compared results in parasite infected (n = 15) and non-infected (n = 36) individuals with a positive QFT-GIT. Though the levels of mRNA for Th1 cytokines were slightly lower in parasite-infected individuals, there was no statistical difference in the level of expression of the selected cytokines between parasite-infected and non-infected groups (Figure 3).

## Conclusions

Latent *M. tuberculosis* infection serves as a reservoir for new cases of TB and better methods of identifying and handling LTBI in TB-endemic countries are urgently needed (for example, active-case-finding) [22]. This has led to interest in comparing results of the TST with the more-specific IGRAs. In this study, we aimed to compare responsiveness to these assays, in a cohort of apparently healthy school children and adolescents, from an area of high TB incidence in Ethiopia. Since currently there is no gold standard to confirm LTBI, the focus has therefore simply been on assay positivity and factors that might affect test results. Although a number of studies have been conducted using whole blood-based IGRAs, of which QuantiFERON-TB Gold is one, to our knowledge this is the first comparative study conducted in Ethiopia on apparently healthy children and adolescents.

In this study, prior testing with tuberculin in at least the past year prior to inclusion to the study was excluded by questionnaire; hence the effect of boosting was assumed to be trivial [23]. The overall rate of reactivity to both TST and QFT-GIT was generally low among the study participants, with 11% reactivity to TST (at a cutoff of 10 mm) and 21% by QFT-GIT. A study conducted in a rural part of Ethiopia, among apparently healthy individuals, also indicated a very low level of reactivity to tuberculin prior to BCG vaccination, which later was boosted after vaccination [24]. On the other hand, a higher TST conversion rate was reported in high-risk groups, living in a suburban area of Ethiopia, though this was at least partly due to BCG vaccination [25]. A very recent report from apparently healthy Ethiopian adults showed a larger proportion of tuberculin reactors [26]. Whether the majority of our participants did not respond to TST because of functionally depressed immune responses or because they were not infected with *M. tuberculosis* cannot be definitively answered by this study. However it can be stated that BCG status had no apparent effect on the degree of concordance between the QFT-GIT and TST results. There was also a trend towards increasing QFT-GIT positivity with age, but this was not seen for the TST. A more detailed analysis of the immunological response in this cohort is underway.

However, the unexpectedly low reactivity to TST, particularly among those who were positive by QFT-GIT, led us to explore the possibility that the TST was in some way inhibited, perhaps by some environmental factors such as parasitic infections. Consistent with this hypothesis, there was a strong association between parasite infestation and a discordant response to testing – in particular with a positive QFT-GIT response and a negative TST (Figure 1). The discordance in this study is primarily associated with results in children from government schools, who were more likely to be QuantiFERON positive than children from private or public schools, but no more likely to be TST+ than children from private schools and significantly less likely to be positive than children from public schools (Table 3). If we look at the population as a whole, at first glance, the TST does not appear to be unduly affected by parasitic infection: 10.2% of students with parasitic infections were TST+, versus 11.1% of students without. In contrast, 31.9% of students with parasitic infections were QFT+ versus 19.1% of students without parasitic infections. The group of students with discordant test results (QFT+/TST-) were disproportionately concentrated in government schools, suggesting that looking at the TST results alone across the whole population may miss some risk factors (there were only two – less than 1% of the students tested - TST+/QFT- discordant results, so we cannot say anything about this group). The mRNA profiles of QFT-GIT+ children in this study matched those previously reported in adult Ethiopians after treatment for TB, or with presumed latent infection [14,20] consistent with the assumption that QFT-GIT+ children probably do represent cases of LTBI (Figure 2). Due to the difficulty of identifying true LTBI, we can do no more than speculate on the relationship of these results to infection at this point, but the results described here suggest that in at-risk populations, the TST may have impaired sensitivity – particularly since a similar profile (higher percentages of QuantiFERON positives than TST positives at a 10 mm cut-off) has also been found in large adolescent cohort studies in India (*Authors, manuscript in preparation*) and South Africa [27].

While the hypothesis that this decreased proportion of TST positives to QFT-GIT positives may be due to co-infection with parasites is attractive (particularly given earlier results from Ethiopia, that deworming improved immune responsiveness to PPD [24,28] this idea must also be approached with caution. It is possible that these infections serve as a proxy for other factors. Parasitic infections were found in roughly double the percentage of students attending government schools (Table 2), compared to private or public schools. But government schools, which are free to attend, tend to attract children from poorer families, who may for example, have nutritional or health deficiencies not identified in our examinations. It should also be noted that a cross-sectional survey like this likely underestimates the prevalence of parasitic infections: many such infections appear to be transient and self-resolving. These results therefore apply to concurrent parasitic infection. We cannot do more than speculate on the potential effect of repeated parasitic infections over time.

In addition, correlations between gut infections and impaired immune responses to PPD or the TST remain controversial: some groups have reported them, others have not [24,28,29]. Some of the differences between reported results are very likely the effect of methodology and population. For example, in a study from Bangladesh, it was suggested that helminth infection could reduce responsiveness in the QFT-GIT [29], in contrast to the results reported here. However, in that study, the difference was seen in an increased number of indeterminate results rather than a decrease in positive results, and malnutrition rather than helminth infection *per se* appeared to be associated with decreased IFN-γ production. In this study, the number of indeterminate results, which can be greatly affected by sample handling, was very low, and malnutrition did not appear to be a significant factor. Consistent with this, parasitic gut infections did not correlate with significantly decreased IFN-γ production (or for that matter increased IL-4 production) in our study population (Figure 2) – a result that also matches with a separate, recent study in pastoralists from Ethiopia [30]. Interestingly, although the focus is different, a study from South Africa looking at atopy noted that a negative TST was associated with increased levels of IgE against the helminth *Ascaris lumbricoides*[31] indicating that there is some degree of interaction. It is likely that multiple factors can modulate these responses, and more research is clearly called for if we are to understand the limits of current immunodiagnostic tools.

## Competing interests

The authors declare that they have no competing interests.

## Authors’ contributions

LW helped design the study, collected data, was involved in data analysis/interpretation and drafted the manuscript. AA, MA, MZG, MZ, AM, GE, MC, HT and LKY were involved in study design, data collection, analysis and write-up. PA was involved in study initiation, and write up of the manuscript. TMD designed the study, and was involved in data analysis, interpretation and write-up. All authors read and approved the final manuscript.

## Pre-publication history

The pre-publication history for this paper can be accessed here:

http://www.biomedcentral.com/1471-2334/13/265/prepub
